# High Resolution Genome Wide Association Studies Reveal Rich Genetic Architectures of Grain Zinc and Iron in Common Wheat (*Triticum aestivum* L.)

**DOI:** 10.3389/fpls.2022.840614

**Published:** 2022-03-16

**Authors:** Jingyang Tong, Cong Zhao, Mengjing Sun, Luping Fu, Jie Song, Dan Liu, Yelun Zhang, Jianmin Zheng, Zongjun Pu, Lianzheng Liu, Awais Rasheed, Ming Li, Xianchun Xia, Zhonghu He, Yuanfeng Hao

**Affiliations:** ^1^Institute of Crop Sciences, Chinese Academy of Agricultural Sciences, Beijing, China; ^2^The Key Laboratory of Crop Genetics and Breeding of Hebei Province, Institute of Cereal and Oil Crops, Hebei Academy of Agricultural and Forestry Sciences, Shijiazhuang, China; ^3^Crop Research Institute, Sichuan Academy of Agricultural Sciences, Chengdu, China; ^4^Research Institute of Grain Crops, Xinjiang Academy of Agricultural Sciences, Urumqi, China; ^5^International Maize and Wheat Improvement Center (CIMMYT) China Office, Beijing, China; ^6^Department of Plant Sciences, Quaid-i-Azam University, Islamabad, Pakistan

**Keywords:** biofortification, candidate genes, GWAS, iron, zinc

## Abstract

Biofortification is a sustainable strategy to alleviate micronutrient deficiency in humans. It is necessary to improve grain zinc (GZnC) and iron concentrations (GFeC) in wheat based on genetic knowledge. However, the precise dissection of the genetic architecture underlying GZnC and GFeC remains challenging. In this study, high-resolution genome-wide association studies were conducted for GZnC and GFeC by three different models using 166 wheat cultivars and 373,106 polymorphic markers from the wheat 660K and 90K single nucleotide polymorphism (SNP) arrays. Totally, 25 and 16 stable loci were detected for GZnC and GFeC, respectively. Among them, 17 loci for GZnC and 8 for GFeC are likely to be new quantitative trait locus/loci (QTL). Based on gene annotations and expression profiles, 28 promising candidate genes were identified for Zn/Fe uptake (8), transport (11), storage (3), and regulations (6). Of them, 11 genes were putative wheat orthologs of known *Arabidopsis* and rice genes related to Zn/Fe homeostasis. A brief model, such as genes related to Zn/Fe homeostasis from root uptake, xylem transport to the final seed storage was proposed in wheat. Kompetitive allele-specific PCR (KASP) markers were successfully developed for two major QTL of GZnC on chromosome arms 3AL and 7AL, respectively, which were independent of thousand kernel weight and plant height. The 3AL QTL was further validated in a bi-parental population under multi-environments. A wheat multidrug and toxic compound extrusion (MATE) transporter *TraesCS3A01G499300*, the ortholog of rice gene *OsPEZ2*, was identified as a potential candidate gene. This study has advanced our knowledge of the genetic basis underlying GZnC and GFeC in wheat and provides valuable markers and candidate genes for wheat biofortification.

## Introduction

Zinc (Zn) and iron (Fe), serving as co-factors for a multitude of enzymes and regulatory peptides in critical metabolic processes, are essential micronutrients for the plant growth and human health (Hänsch and Mendel, 2009). Due to monotonous diet and heavy relying on cereal edible parts with suboptimal micronutrient levels, Zn and Fe deficiencies have become the most common public health problem in the world, especially for pregnant women and young children due to their increased demands for micronutrients (Bhati et al., 2016; Vasconcelos et al., 2017). Increasing intrinsic micronutrients in the edible parts of crops, known as biofortification, is regarded as the most cost-effective and sustainable intervention to alleviate Zn and Fe malnutrition in humans (Gómez-Galera et al., 2010). Common wheat (*Triticum aestivum* L.) as a staple food crop, supplying approximately 20% of daily calories and protein, and main source of essential micronutrients, such as Zn and Fe, is recognized as an attractive crop for biofortification (Ludwig and Slamet-Loedin, 2019). For nutritionally sufficient wheat Zn and Fe biofortification, the concentrations of Fe and Zn in whole grains have to, respectively, reach 37 and 59 mg/kg, about 50% higher than the average concentrations of popular wheat cultivars (Bouis et al., 2011). However, breeding elite cultivars with enhanced Zn/Fe content is quite challenging due to the obscurity of genetic architecture and molecular processes regulating the Zn/Fe homeostasis in wheat, which greatly hampers the implements of modern breeding technologies, such as marker-assisted selection (MAS) and genomic selection (GS) (Gupta et al., 2021).

To improve our understanding of the genetic basis of wheat grain Zn and Fe, identification of as many causal loci as possible is imperative (Tong et al., 2020). In recent years, diverse bi-parental populations have been used to identify quantitative trait locus/loci (QTL) associated with grain Zn (GZnC) and Fe concentrations (GFeC) in common wheat and its relative species (Tong et al., 2020; Gupta et al., 2021). These QTL from different studies have been integrated into a consensus map according to the physical positions of their linked markers, and provide a valuable resource for dissection of the genetic architecture underlying GZnC and GFeC (Tong et al., 2020). Nevertheless, the family based bi-parental populations usually have limited number of recombination events and low genetic diversity, thus low-mapping resolution and may be unable to provide a full genome-wide genetic landscape of complex traits (Korte and Farlow, 2013; Platten et al., 2019). As a complementary strategy to QTL mapping, a genome-wide association study (GWAS) is a powerful tool to detect the genetic regions underlying complex traits using the historical abundant crossovers and genetic variations accumulated in natural wheat germplasms (Hamblin et al., 2011). A number of genetic loci associated with GZnC and GFeC have been identified by GWAS in wheat recently (Tong et al., 2020; Gupta et al., 2021). However, large gaps in the genetic map due to low marker number and density were generally found, which greatly hindered precision in the dissection of the GZnC and GFeC traits (Korte and Farlow, 2013).

With the rapid development of next-generation sequencing (NGS) technology, high-density single nucleotide polymorphism (SNP) genotyping arrays have been developed in wheat (reviewed in Rasheed et al., 2017). For example, the Wheat Axiom 660K SNP array with more than half a million markers enables high marker resolution, large genome coverage, and low heterozygosity (Sun et al., 2020). GWAS using such high density SNPs will make it possible to obtain a large number of associated loci within very small intervals and consequently facilitate the candidate gene discovery and genetic dissection of complex traits (Yano et al., 2016; Pang et al., 2020). The mixed linear model (MLM), the fixed and random model circulating probability unification (FarmCPU), and the multiple loci mixed linear model (MLMM) in different strengths for each have been adopted to effectively control the population structure and to ensure the accuracy and reliability of significantly associated loci. It is appropriate to adopt multiple models simultaneously to conduct the GWAS for genetic dissection of complex traits (Peng et al., 2018; Alqudah et al., 2020).

In the current study, a diverse panel of 166 representative wheat accessions was chosen from elite germplasm and was genotyped with the wheat 660K and 90K SNP arrays. GWASs were carried out using three different models for GZnC and GFeC across multiple environments. Furthermore, plant height (PH), thousand kernel weight (TKW), and grain areas (GA) were investigated to study their associations with GZnC/GFeC and identify pleiotropic loci. The study aimed to (1) dissect the genetic architecture of GZnC and GFeC, (2) identify associated markers, loci, and candidate genes, and (3) develop high-throughput kompetitive allele-specific PCR (KASP) markers and validate the major QTL for wheat biofortification.

## Materials and Methods

### Plant Material and Field Trials

The association panel consists of 166 representative wheat cultivars, such as 144 accessions from Yellow and Huai Valley, the major wheat zone in China, and 22 from five other countries (Supplementary Table 1). The panel was used in our previous studies on black point (Liu et al., 2017), flour color and polyphenol oxidase (PPO) activity (Zhai et al., 2018, 2020), grain yield related traits (Li et al., 2019), and water-soluble carbohydrate contents (Fu et al., 2020). In this study, the panel was grown in randomized complete blocks with two replications in four environments comprising Beijing (39°56′N, 116°20′E), Gaoyi (37°33′N, 114°26′E), and Shijiazhuang (37°27′N, 113°30′E) in Hebei province, and Urumqi (42°45′N, 86°37′E) in Xinjiang province during the 2019–2020 cropping season. These environments were designated as 20BJ, 20GY, 20SJZ, and 20XJ hereafter, respectively. Each entry with approximately 40–50 grains was grown in 1.0 m long row with an inter-row spacing of 20.0 cm. Standard agronomic practices were performed at each location, along with soil application of 25 kg/ha ZnSO_4_.7H_2_O granular fertilizer (Sinochem Group Co., Ltd., Beijing, China) in 20GY and 20SJZ over three crop cycles prior to this experiment to enrich the available soil Zn and minimize heterogeneity. The green manure returning was routinely practiced in Beijing and field soil held sufficient Zn intrinsically, so that additional Zn fertilizer was not applied in 20BJ. To investigate GZnC and GFeC in a low-Zn environment, 20XJ was set without soil Zn fertilizer application.

### Phenotype Determination and Statistical Analyses

Plant materials were hand-harvested in the field and completely dried grains were hand-threshed and cleaned carefully to avoid the potential contamination of mineral element. Approximately 15 g of each sample was subjected to GZnC and GFeC analyses with an X-ray fluorescence spectrometry (EDXRF) instrument (model X-Supreme 8000, Oxford Instruments plc.), following the protocol of high-throughput screening of micronutrients in whole-wheat grain (Paltridge et al., 2012). PH, TKW, and GA in all environments were investigated following Xu et al. (2019).

ANOVA, Pearson’s correlation analysis, and Student’s *t*-test were conducted using the SAS 9.4 software (SAS institute, Cary, NC, United States). Broad-sense heritabilities (*H*^2^) were calculated using the following equation (Holland et al., 2003):


H=2σg2/(σg2+σge2/e+σε2/(re))

where σ*^2^_*g*_* indicates the variance of genotypes, σ*^2^_*ge*_* and σ*^2^_ε_* represent the variances of genotype × environment interaction and errors, and *e* and *r* are the numbers of environments and replicates in each environment, respectively. For each trait, a best linear unbiased estimation (BLUE) using a linear model for each accession was calculated across environments by the QTL IciMapping v4.1 software (Yin et al., 2015).

### Genotyping and Physical Map Construction

Genomic DNA was extracted following the CTAB method (Murray and Thompson, 1980) and the population was genotyped by the Illumina wheat 90K (such as, 81,587 SNPs) and Affymetrix wheat 660K (such as, 630,517 SNPs) SNP arrays by CapitalBio Technology Co., Ltd, Beijing, China.^1^ Markers with minor allele frequency (MAF) < 5.0% and missing data containing heterozygous genotypes > 20.0% were filtered out and the same final 373,106 high-quality polymorphic SNPs were obtained (Fu et al., 2020). These markers included 359,760 (96.4%) from the 660K and 13,346 (3.6%) from 90K SNP array, indicating SNPs in wheat 90K array have relatively low polymorphisms in Chinese cultivars. Flanking sequences of SNP markers were used for BLAST analysis against IWGSC RefSeq v.1.0,^2^ to obtain physical positions in accordance with the best BLAST hit results. Eventually these markers were integrated into one consensus map, covering a total physical distance of 13.7 Gb, accounting for about 95% of wheat whole genome sequences, and were further utilized for GWAS. Relevant information is available in our previous studies (Liu et al., 2017; Fu et al., 2020).

### Genome-Wide Association Study

The 166 accessions were classified into three subpopulations by the population structure using Structure v2.3.4 (Liu et al., 2017). The average linkage disequilibrium (LD) decay distances for A, B, D, and whole genomes were approximately 6, 4, 11, and 8 Mb, respectively (Liu et al., 2017). In this study, GWAS was conducted for each environment and BLUE values of GZnC across 20GY, 20SJZ, and 20BJ. The site 20XJ was excluded from the analysis owning to relatively low Pearson’s correlation coefficients with other environments (data not shown). The low Pearson’s correlation was possibly attributed to soil Zn content heterogeneity in 20XJ with null Zn fertilizer application (Velu et al., 2018). As for GFeC, GWAS was carried out across all four environments and BLUE values. Three models, MLM (*Q* + *K*), FarmCPU, and MLMM, were employed for the GWAS in GAPIT a software package operating in R v3.5.1^3^ (Tang et al., 2016). To maximize chances of identifying all possible QTL, a threshold of *p* = 1.0 × 10^–3^ (−log_10_ (*p*) = 3.0) was set for the significance of marker-trait associations (MTAs). To control the false discovery rate (FDR) at an appropriate level, those detected in three or more environments (BLUE value was regarded as one environment hereafter) by any of the three models were considered as reliable MTAs (Fu et al., 2020). MTAs detected in the same LD block or physically closely linked were grouped into a single QTL, and the distance between the two very far flanking markers was seen as the QTL interval. The most significant MTA with the lowest value of *p* across environments in this interval was selected as the representative marker, and its *R*^2^ output by MLM was used to reflect the proportion of phenotypic variance explained (Fu et al., 2020). Manhattan and quantile–quantile (*Q*–*Q*) plots were generated using the CMplot package in R v3.5.1 software.^4^

### Candidate Gene Identification

According to the gene annotations from IWGSC RefSeq v.1.0 and putative homologs in the UniProt database,^5^ the genes located in or adjacent to the physical intervals of QTL identified in this study were subjected to be screened as follows. (1) Those related to the molecular processes in Zn/Fe homeostasis, such as metal uptake in the root, transport in the xylem and phloem, storage in seeds, and regulatory factors, were predicted to be potential candidates; (2) analysis of orthologs between wheat and model plants was carried out to obtain candidate genes in Triticeae-Gene Tribe browser^6^ (Chen et al., 2020) with most of the genes collected in Tong et al. (2020); (3) haplotype analysis was performed within Wheat SnpHub Portal^7^ for 641 accessions with known genome sequences (Hao et al., 2020; Wang et al., 2020), and the genes without sequence variation were removed from candidacy. The most promising candidate genes were eventually selected. The database expVIP^8^ was used to investigate spatio-temporal transcriptional dynamics of candidate genes, providing expression profiles of these genes across tissues and developmental stages (Ramírez-González et al., 2018).

### Kompetitive Allele-Specific PCR Marker Development and Quantitative Trait Locus/Loci Validation

The representative markers for 3AL and 7AL QTL were chosen and converted to KASP markers. Chromosome-specific primers were designed using Polymarker^9^ (Ramirez-Gonzalez et al., 2015) and KASP assays were performed following Xu et al. (2020). The KASP markers were successfully converted when their genotypic results were the same as the original SNP arrays. The 146 F_6_ recombinant inbred lines (RILs) developed from a cross between two modern wheat cultivars Zhongmai 175 and Lunxuan 987 (shorten as ZM175/LX987) with significantly different GZnC were used as validation population for the target QTL. GZnC was determined in the bi-parental population in the same way as mentioned earlier in the natural population. Student’s *t*-tests were conducted to verify allelic effects based on the phenotypic data from the bi-parental population. For the candidate gene of QTL, its variations between ZM175 and LX987 were extracted from the genome resequencing database at Wheat SnpHub Portal (see text footnote 7; Hao et al., 2020; Wang et al., 2020).

## Results

### Phenotypic Variations

A wide range of continuous variations of GZnC and GFeC were observed for the 166 accessions across all environments with near-normal distributions (Supplementary Figures 1, 2). The BLUE values for GZnC and GFeC were 29.25–50.98 mg/kg and 39.86–54.66 mg/kg with mean values of 38.03 and 45.97 mg/kg, respectively (Table 1). In all the accessions, six cultivars with the highest values for GZnC and GFeC were selected with the BLUE values greater than 50 mg/kg and stable performance across the environments (Supplementary Figure 3). Yumai2 and Xinong1376 were the cultivars with both high GZnC and GFeC. Significant correlations (*r*) between environments were observed for both GZnC and GFeC (Supplementary Figure 4). The results of ANOVA revealed that the factors of genotypes, environments, and G × E interactions markedly affect the GZnC and GFeC (Supplementary Table 2). Broad sense heritabilities (*H*^2^) of GZnC and GFeC were 0.71 and 0.72, respectively, indicating a determinant role of genetic factors for phenotypic variations (Table 1). A significant and positive correlation was found between GZnC and GFeC based on BLUE values (*r* = 0.45, *p* < 0.0001). GFeC was positively correlated with PH (*r* = 0.35, *p* < 0.0001), whereas GZnC was not (Supplementary Figure 4). TKW was positively correlated with GA and negatively correlated with PH, and appeared not to be correlated with either GZnC or GFeC.

**TABLE 1:** The phenotypic variation and *H*^2^ of GZnC and GFeC in 166 wheat cultivars across different environments^a^.

Trait	Environment	Min (mg/kg)	Max (mg/kg)	Mean (mg/kg)	SD (mg/kg)	*H* ^2^
GZnC	20BJ	28.60	54.30	37.38	4.07	0.71
	202GY	31.80	58.90	43.68	5.92	
	20SJZ	21.80	52.00	33.02	5.53	
	BLUE	29.25	50.98	38.03	3.95	
GFeC	20BJ	32.50	57.70	41.51	4.39	0.72
	20GY	35.20	60.95	45.96	4.80	
	20SJZ	36.00	57.50	43.82	4.27	
	20XJ	39.70	74.60	52.60	5.79	
	BLUE	39.86	54.66	45.97	3.56	

*^a^GZnC, grain zinc concentration; GFeC, grain iron concentration; 20BJ, 20GY, 20SJZ, 20XJ: Beijing, Gaoyi, Shijiazhuang, and Xinjiang locations, respectively, 2019–2020; BLUE: best linear unbiased estimation; SD, standard derivation; H^2^, broad-sense heritability.*

### Marker-Trait Associations and Pleiotropic Loci

**FIGURE 1:**
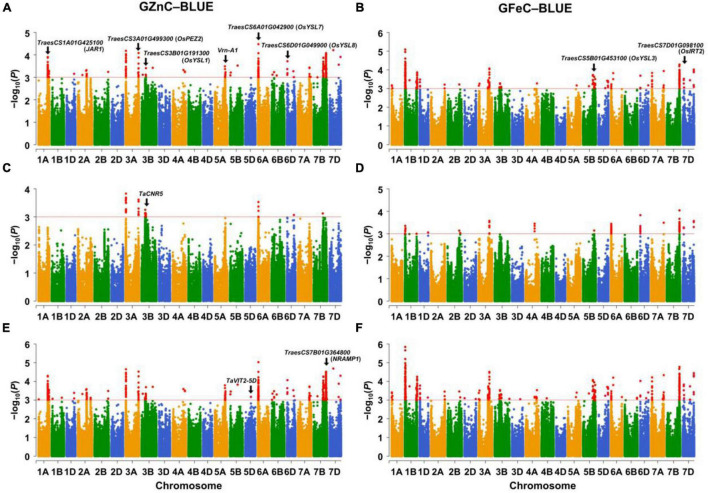
Manhattan plots for GZnC and GFeC analyzed by **(A,B)** the mixed linear model (MLM), **(C,D)** the fixed and random model circulating probability unification (FarmCPU), and **(E,F)** the multiple loci mixed linear model (MLMM). The threshold of *p* = 1.0 × 10^–3^ (–log_10_ (*p*) = 3.0) was used for calling significant marker-trait associations (MTAs). GZnC and GFeC indicate grain zinc and iron concentrations, respectively. BLUE indicates the best linear unbiased estimations across environments. The wheat gene ID indicates cloned wheat genes or wheat orthologs of known Zn/Fe-related genes in model plants.

The significant associations were identified between SNPs and GZnC and GFeC using MLM, FarmCPU, and MLMM based on the BLUE values (Figure 1) and the values in each environment (Supplementary Figures 5A, 6A). The *Q*–*Q* plots showed that the observed values of *p* were close to the expected distributions indicating the proper control of false positive in GWAS (Supplementary Figures 5B, 6B). Among the 2,214 and 1,340 significant MTAs for GZnC and GFeC, respectively, 154 and 72 corresponding to 25 and 16 different loci were identified in at least three environments. These 41 loci were considered as stable QTL and are summarized in Table 2. In total, 9, 11, and 8 QTL were only detected by MLM, MLMM, and FarmCPU, respectively; five QTL were identified by two models; eight were simultaneously detected through all the three models (Supplementary Figure 7), indicating the complementarity and reliability of the three models we employed.

**TABLE 2:** Significant loci associated with GZnC and GFeC in at least three environments in 166 wheat accessions using three models in GAPIT.

Trait^a^	Environment^b^	Chr^c^	Physical interval (Mb)^d^	Representative SNP^e^	Allele^f,g^	MAF^f,h^	*P*-value (× 10^–4^)^f^	*R*^2^ (%)^f,i^	Effect^f^	*P*-value for PH^j^	*P*-value for TKW^k^	Reported QTL^l^
GZnC	E1, E2, BLUE	1A	515.79–516.03	*AX_108845138*	C/T	0.11	1.11	11.88	2.58	0.439	0.765	*QGZn.cimmyt-1A*
	E2, E3, BLUE	1A	580.42–586.13	*AX_109976007*	C/G	0.44	2.36	9.62	2.25	0.935	0.461	*QGZn.cau-1A*
	E2, E3, BLUE	2A	246.51	*AX_109885783*	C/G	0.13	5.87	8.55	2.87	0.883	0.867	
	E2, E3, BLUE	3A	10.22	*AX_111534768*	G/A	0.18	1.91	9.91	3.19	0.964	0.164	
	E1, E2, E3, BLUE	3A	19.89–22.61	*AX_111528452*	T/G	0.31	0.15	13.57	3.06	0.377	0.001	
	E2, E3, BLUE	3A	696.23	*AX_108843516*	C/A	0.44	4.82	8.62	2.19	0.489	0.585	*QGZn.cimmyt-3A.1*
	E2, E3, BLUE	3A	721.64–724.58	*AX_109875082*	T/A	0.46	0.20	13.53	2.63	0.061	0.850	*QGZn.cimmyt-3A.2*
	E2, E3, BLUE	3B	202.41	*AX_110479767*	A/G	0.19	5.69	8.39	5.73	0.482	0.698	
	E2, E3, BLUE	3B	242.66	*AX_109384871*	G/A	0.17	7.35	8.04	6.80	0.177	0.527	
	E2, E3, BLUE	5A	591.28–592.63	*AX_94729189*	G/C	0.24	1.96	10.11	2.46	0.075	0.063	
	E2, E3, BLUE	5D	319.22	*AX_94388289*	C/G	0.20	4.21	8.81	2.82	0.284	0.599	
	E2, E3, BLUE	6A	17.77	*AX_110365398*	A/G	0.36	6.80	8.20	1.76	0.831	0.872	
	E1, E2, E3, BLUE	6A	28.31–31.37	*AX_111556928*	A/G	0.16	0.18	10.03	2.67	0.813	0.397	
	E2, E3, BLUE	6B	142.73	*AX_110935664*	A/G	0.39	0.79	11.44	2.41	0.391	0.830	*QGZn.uh-6B*
	E1, E2, BLUE	6D	16.8–16.81	*AX_108846745*	T/C	0.49	4.32	8.83	1.75	0.602	0.507	
	E2, E3, BLUE	6D	27.52	*AX_95220141*	G/A	0.20	1.45	10.55	3.19	0.554	0.422	
	E2, E3, BLUE	6D	357.01	*AX_110431664*	T/C	0.25	2.78	9.38	2.81	0.008	0.706	
	E1, E2, BLUE	7A	706.91	*AX_94741862*	T/G	0.35	9.57	7.73	1.64	0.205	0.343	*QGZn.ua-7A*
	E1, E2, E3, BLUE	7B	533.78–540.78	*Tdurum_contig65979_ 289*	A/G	0.39	1.34	10.48	1.97	0.920	0.974	
	E1, E2, E3, BLUE	7B	626.06–626.07	*BS00022045_51*	A/G	0.48	0.52	13.09	1.66	0.091	0.524	*QGZn.cimmyt-7B.5*
	E1, E2, E3, BLUE	7B	687.32–689.92	*AX_110464521*	G/A	0.26	0.76	12.48	1.75	0.708	0.491	
	E1, E2, BLUE	7B	708.11	*AX_95000860*	T/C	0.49	1.97	10.98	1.51	0.716	0.918	*GZn-IWA4150*
	E2, E3, BLUE	7D	203.16	*AX_110717434*	C/T	0.14	0.19	13.24	3.83	0.231	0.891	
	E1, E3, BLUE	7D	506.11	*GENE_3452_1116*	G/A	0.38	2.74	9.47	1.86	0.485	0.399	
	E1, E2, E3, BLUE	7D	605.17	*AX_108866365*	C/G	0.19	1.24	10.60	2.16	0.697	0.812	
GFeC	E2, E3, BLUE	1A	15.43	*AX_95081354*	C/T	0.20	5.43	8.37	2.07	0.047	0.102	
	E1, E2, BLUE	1B	15.65–15.74	*AX_86185361*	C/G	0.30	2.03	9.12	1.72	0.222	0.182	
	E2, E4, BLUE	1B	26.17–26.37	*AX_111633663*	A/C	0.45	0.11	13.03	2.01	0.007	0.246	
	E1, E4, BLUE	1B	38.63–38.83	*AX_109837760*	T/G	0.48	0.45	11.13	1.71	0.002	0.742	*QGFe.cimmyt-1B*
	E2, E3, BLUE	1B	660.01	*Tdurum_contig68980_ 448*	A/G	0.29	4.96	7.97	1.43	0.202	0.018	
	E1, E4, BLUE	1B	688.28–689.27	*AX_110457631*	C/T	0.36	1.37	9.64	1.42	0.087	0.618	
	E1, E4, BLUE	5A	495.92	*AX_109311262*	G/A	0.09	6.82	7.56	2.01	0.000	0.331	*QGFe.sau-5A.1*
	E2, E3, BLUE	5B	531.58	*AX_94967094*	C/T	0.39	5.82	7.76	1.39	0.668	0.480	*QGFe.cimmyt-5B.2*
	E2, E3, BLUE	5B	548.33	*AX_111484713*	G/A	0.20	1.89	9.22	1.68	0.404	0.002	
	E2, E3, BLUE	5B	622.54	*AX_111033847*	C/T	0.32	2.38	8.92	1.50	0.097	0.176	*QGFe.cimmyt-5B.3*
	E3, E4, BLUE	5B	679.03	*AX_109412899*	G/A	0.18	3.13	8.56	1.69	0.696	0.163	*QGFe.cimmyt-5B.4*
	E3, E4, BLUE	7A	706.91	*AX_94741862*	T/G	0.35	1.19	9.82	1.38	0.196	0.165	*QGFe.iari-7A*
	E3, E4, BLUE	7B	706.37–706.86	*Tdurum_contig61856_ 900*	A/C	0.36	0.52	10.93	1.42	0.001	0.035	*QGFe.cimmyt-7B*
	E2, E3, BLUE	7D	54.99	*AX_108920250*	C/T	0.44	0.14	14.49	2.27	0.989	0.005	
	E2, E3, BLUE	7D	69.31	*AX_111359934*	G/A	0.40	4.67	8.58	1.65	0.937	0.142	
	E1, E2, E3, E4, BLUE	7D	614.51–614.92	*AX_95151824*	T/G	0.32	0.96	10.12	1.43	0.122	0.101	*QGFe.sau-7D*

*^a^GZnC, grain zinc concentration; GFeC, grain iron concentration.*

*^b^E1, E2, E3, E4: Beijing, Gaoyi, Shijiazhuang, and Xinjiang locations, respectively, 2019–2020; BLUE, best linear unbiased estimation; BLUE-value was also used to conduct GWAS and was regarded as one environment.*

*^c^Chr, chromosome.*

*^d^Physical positions of single nucleotide polymorphism (SNP) markers were based on IWGSC RefSeq v.1.0 (http://www.wheatgenome.org/).*

*^e^The most significant SNP with the lowest p across environments for the corresponding locus was regarded as a representative.*

*^f^The information in corresponding columns are based on the representative SNP.*

*^g^“_” indicates the favorable allele with the increasing effect on GZnC or GFeC.*

*^h^MAF: minor allele frequency.*

*^i^R^2^ indicates the percentage of phenotypic variance explained by the SNP marker.*

*^j^PH, plant height. The values of p for association between the representative markers of GZnC/GFeC QTL and plant height were calculated by GAPIT using the MLM model.*

*^k^TKW, thousand kernel weight. The p for association between the representative markers of GZnC/GFeC QTL and thousand kernel weight were calculated by GAPIT using the MLM model.*

*^l^The closest linked markers or mid-points of previous reported QTL intervals are present in Tong et al. (2020). Those loci with physical distances smaller than or approximate to one LD block away from reported QTL were considered as the same with the previous QTL.*

For GZnC, 25 stable loci located on chromosomes 1A (2), 2A, 3A (4), 3B (2), 5A, 5D, 6A (2), 6B, 6D (3), 7A, 7B (4), and 7D (3) explained the phenotypic variation (*R*^2^-value) ranging from 7.73 to 13.57% (Table 2). Among these, six loci on chromosomal arms 3AS (*AX_111528452*, 19.89 Mb), 6AS (*AX_111556928*, 30.88 Mb), 7BL (*Tdurum_contig65979_289*, 539.22 Mb; *BS00022045_51*, 626.07 Mb; *AX_11046452*, 687.84 Mb), and 7DL (*AX_108866365*, 605.17 Mb) were the most stable and identified in all environments. The most significant marker was *AX_111528452* on 3AS with a *p* at 1.5 × 10^–5^ and *R*^2^-value at 13.57%. For GFeC, we detected 16 stable QTL on chromosomes 1A, 1B (5), 5A, 5B (4), 7A, 7B, and 7D (3) with *R*^2^-values ranging from 7.56 to 14.49% (Table 2). One stable locus on the long arm of chromosome 7D (*AX_95151824*, 614.54 Mb) was detected in all five environments comprising BLUE values. The most significant QTL was identified on 1BS (*AX_111633663*) with a *p* at 1.1 × 10^–5^ in the genomic region of 26.17–26.37 Mb.

Four pleiotropic loci were identified by comparing their physical positions of stable MTAs for GZnC, GFeC, and PH (Table 2 and Supplementary Table 3). One locus on 7AL (*AX_94741862*, 706.91 Mb) was simultaneously associated with GZnC and GFeC while independent of PH and TKW, indicating its potential value in breeding for high grain zinc and iron. Two loci on 5AL (*AX_109311262*, 495.92 Mb) and 7BL (*Tdurum_contig61856_900*, 706.45 Mb) significantly increased GFeC and PH, suggesting that they might be useful when plant height is not an issue. The fourth pleiotropic locus located on 3AS (*AX_111528452*, 20.33 Mb) increased the GZnC but decreased the TKW. All the other 37 MTAs identified have no obvious pleiotropic effects and can be easily used in breeding for genetic improvement of either GZnC or GFeC (Table 2).

### Candidate Genes Underlying Stable Loci

Twenty-eight promising candidate genes, located in or adjacent to the physical intervals of the QTL, were identified to be potentially involved in Zn/Fe uptake, transport, storage, and regulations (Table 3). They showed polymorphisms in 641 cultivars which were re-sequenced (data now shown). Among them, 17 genes were located within 2 Mb proximity of the representative markers, and nine genes were located less than 1 Mb from the marker (Table 3). The remaining 11 genes, mainly cloned wheat genes or wheat orthologs of known genes in model plants (7 out of 11), were likely to be causal genes for Zn/Fe homeostasis.

**TABLE 3:** Putative candidate genes underlying the loci associated with GZnC and GFeC.

Trait^a^	Chr^b^	Physical interval of identified QTL (Mb)^c^	Candidate gene^d^	Physical position^c^	Distance^d^ (Mb)	Ortholog/Putative functionality^e^	Putative involved process^f^
GZnC	1A	515.79–516.03	*TraesCS1A01G326700*	516.73	0.70	Citrate-binding protein	Transport
	1A	580.42–586.13	*TraesCS1A01G425100*	580.18	5.95	*JAR1* (Kobayashi, 2019)	Regulations
	3A	19.89–22.61	*TraesCS3A01G036400*	19.98	0.09	*ABC* transporter G family member	Uptake
	3A	721.64–724.58	*TraesCS3A01G499300*	724.49	0.09	*OsPEZ2* (Bashir et al., 2011; Ishimaru et al., 2011)	Uptake
	3B	202.41	*TraesCS3B01G191300*	204.28	1.88	*OsYSL1* (Chu et al., 2010)	Transport
	3B	242.66	*TraesCS3B01G214000*	253.90	11.24	*TaCNR5* (Qiao et al., 2019)	Transport
	5A	591.28–592.63	*TraesCS5A01G391700*	587.40	5.24	*Vrn-A1* (Jobson et al., 2018)	Regulations
	5D	319.22	*TraesCS5D01G209900*	318.10	1.12	*TaVIT2-5D* (Connorton et al., 2017)	Storage
	6A	17.77	*TraesCS6A01G042900*	22.54	4.76	*OsYSL7* (Chu et al., 2010)	Transport
	6A	28.31–31.37	*TraesCS6A01G051700*	26.93	1.95	*NAC* domain-containing protein	Regulations
	6B	142.73	*TraesCS6B01G145600*	145.88	3.14	*ABC* transporter B family protein	Uptake
	6D	27.52	*TraesCS6D01G049900*	24.19	3.33	*OsYSL8* (Grillet and Schmidt, 2019)	Transport
	7A	706.91	*TraesCS7A01G527900*	708.67	1.76	Magnesium transporter	Uptake
	7B	533.78–540.78	*TraesCS7B01G299200*	536.01	3.21	*BZIP* transcription factor	Regulations
	7B	626.06–626.07	*TraesCS7B01G364800*	627.98	1.91	*NRAMP1* (Segond et al., 2009)	Storage
	7B	687.32–689.92	*TraesCS7B01G429600*	698.14	9.30	Zinc ion binding protein	Storage
	7B	708.11	*TraesCS7B01G444600*	708.95	0.83	Magnesium transporter	Transport
	7D	203.16	*TraesCS7D01G237500*	201.88	1.29	Copper-transporting ATPase	Uptake
	7D	506.11	*TraesCS7D01G392800*	507.81	1.70	*BZIP* transcription factor	Regulations
	7D	605.17	*TraesCS7D01G504400*	610.53	5.36	Heavy metal transport	Transport
GFeC	1B	15.65–15.74	*TraesCS1B01G030800*	15.16	0.28	*ABC* transporter ATP-binding protein	Uptake
	1B	660.01	*TraesCS1B01G437100*	659.98	0.03	Calcium-transporting ATPase	Uptake
	5B	531.58	*TraesCS5B01G349500*	530.78	0.80	Fe^2+^ transporter	Transport
	5B	548.33	*TraesCS5B01G370100*	548.81	0.47	ATP-dependent zinc metalloprotease	Transport
	5B	622.54	*TraesCS5B01G453100*	626.01	3.46	*OsYSL3* (Waters et al., 2006)	Transport
	7D	54.99	*TraesCS7D01G098100*	58.90	3.90	*OsIRT2* (Ishimaru et al., 2006)	Uptake
	7D	69.31	*TraesCS7D01G113100*	69.26	0.06	*BHLH* family transcription factor	Regulations
	7D	614.51–614.92	*TraesCS7D01G515800*	615.91	1.37	Magnesium transporter	Transport

*^a^GZnC, grain zinc concentration; GFeC, grain iron concentration.*

*^b^Chr, chromosome.*

*^c^Physical positions of SNP markers and annotated genes were based on IWGSC RefSeq v.1.0.*

*^d^The distances between representative markers and candidate genes.*

*^e^Gene ID and functional annotations were based on IWGSC RefSeq v.1.0. The underlined genes indicated the cloned Zn/Fe-related genes in common wheat.*

*^f^Putative involved processes were based on the regulatory mechanisms of corresponding orthologous gene in Arabidopsis and rice, as well as gene annotations from IWGSC RefSeq v.1.0.*

For Zn/Fe uptake, generally proton ATPases, nicotianamine synthases, and phenolic compound transporter played key roles. At least eight candidate genes were identified, and two of them, *TraesCS3A01G499300* and *TraesCS7D01G098100*, were wheat orthologs of rice genes *OsPEZ2* (Bashir et al., 2011; Ishimaru et al., 2011) and *OsIRT2* (Ishimaru et al., 2006) with known functions for Zn/Fe homeostasis. The expression profiles showed that all the above genes expressed at high levels in roots, further supporting their potential roles in Zn/Fe acquisition (Supplementary Figure 8). For Zn/Fe translocation, key transporters, such as citrate efflux transporter, yellow stripe-like (*YSL*) transporter, zinc-regulated transporter, and iron-regulated transporter-like protein (*ZIP*) transporter, were predominantly responsible for this process, and 11 putative genes were identified accordingly. Four genes were wheat orthologs of *YSL* genes (*OsYSL1*, *OsYSL3*, *OsYSL7*, and *OsYSL8*) in rice (Waters et al., 2006; Chu et al., 2010; Grillet and Schmidt, 2019), one was cloned gene (*TaCNR5*) in wheat (Qiao et al., 2019) related to zinc homeostasis, and the remaining six were possible wheat specific or yet to be discovered in model plants. These genes showed diverse expression patterns in different tissues and ages (Supplementary Figure 8). None or very little expression was detected in wheat grain, which partially supported their Zn/Fe transport roles in vegetative tissues (Kobayashi and Nishizawa, 2012).

The storage of metal into vacuoles in seeds was mainly mediated by vacuolar iron transporter (*VIT*) family members (Zhang et al., 2012), and the efflux of metal from the vacuolar to cytosol was mainly controlled by *NRAMP* family (Segond et al., 2009), which made *TraesCS5D01G209900* (*TaVIT2-5D*) and *TraesCS7B01G364800* (ortholog of *AtNRAMP1*) promising candidate genes responsible for the Zn/Fe storage in seeds (Segond et al., 2009; Connorton et al., 2017). *TraesCS7B01G429600* encoding a zinc binding protein showed high expression in wheat grain and was also considered as a promising candidate gene involved in the Zn/Fe storage (Supplementary Figure 8). Additionally, six regulator genes, such as *Vrn-A1*, mainly known as transcriptional factors (TFs) regulating the plant development, were identified. *TraesCS7B01G299200* and *TraesCS7D01G392800*, the *bZIP* TFs, *TraesCS7D01G113100*, the *bHLH* TF, *TraesCS6A01G0517000*, the NAC domain protein, and *TraesCS1A01G425100*, the wheat ortholog of jasmonic acid-amido synthetase (*JAR1*), were all putatively related to Zn/Fe homeostasis in wheat. Their spatio-temporal expression profiles are provided in Supplementary Figure 8.

### Validation of 3AL Quantitative Trait Locus/Loci and the Underlying Gene

**FIGURE 2:**
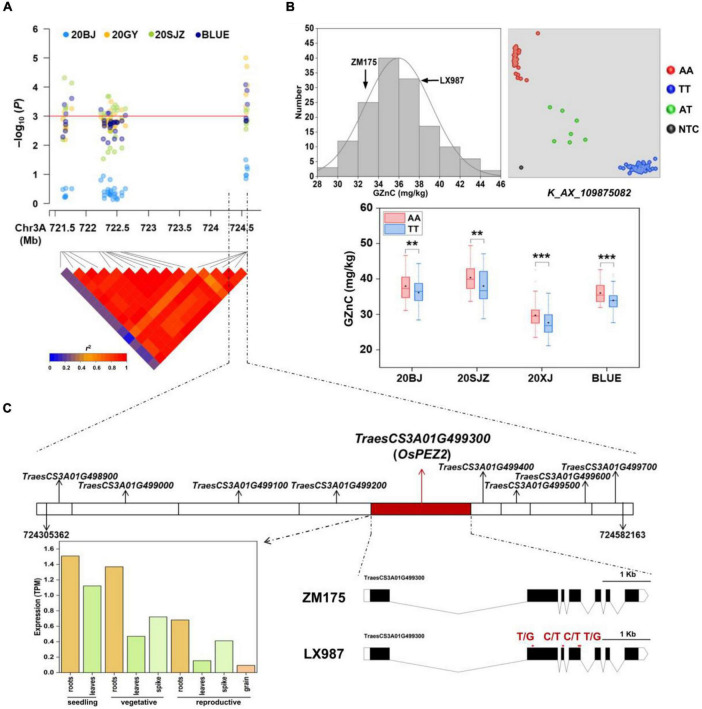
**(A)** Local Manhattan plot and linkage disequilibrium (LD) heatmap of single nucleotide polymorphisms (SNPs) within the 3AL quantitative trait loci (QTL) identified for grain zinc concentration (GZnC). 20BJ, 20GY, and 20SJZ: Beijing, Gaoyi, and Shijiazhuang locations, respectively, 2019–2020. BLUE: best linear unbiased estimations. **(B)** The validation of 3AL QTL in ZM175/LX987 RIL population. Histograms for GZnC in the population using BLUE values across three environments (top and left); genotype calling result of the kompetitive allele-specific PCR (KASP) markers for *K_AX_109875082* in the population (top and right); allelic effects of *K_AX_109875082* on GZnC in the population across 20BJ, 20SJZ, 20XJ, and BLUE (bottom). AA and TT indicate two homozygous genotypes for this marker from LX987 and ZM175, respectively. AT indicates the heterozygous genotype, and NTC represents no template control. 20XJ: Xinjiang location in during 2019–2020. The black diamond in each box indicates the mean. *p < 0.05; **p < 0.01; ***p < 0.001; ns: not significant. **(C)** The nine annotated genes identified close to *AX_109875082* with the highest *p* in the 3AL QTL region (top); the spatio-temporal expression profiles of the candidate gene *TraesCS3A01G499300* (bottom and left); the gene structure of *TraesCS3A01G499300* containing four exons with missense variants between ZM175 and LX987 (bottom and right), and red arrows indicate the positions of the exon missense variants between ZM175 and LX987.

Among all the MTAs, we chose the major QTL on 3AL for GZnC (*AX_109875082*, 724.58 Mb) with high *R*^2^-value (13.53%) and the pleiotropic locus on 7AL for GZnC and GFeC (*AX_94741862*, 706.91 Mb) to develop high-throughput KASP markers (Table 2 and Supplementary Table 4). Both QTL were independent of TKW and PH (Table 2). Their representative SNPs were successfully converted to KASP assays after genotyping the association panel and the same genotypic data were obtained from the wheat 660K SNP array (data not shown). The marker information was presented in Supplementary Table 4. The two KASP markers were further used to test the ZM175/LX987 bi-parental population. For *K*_*AX_109875082* on 3AL, a *t*-test showed a significant difference of GZnC between lines carrying favorable allele and lines with opposite allele across environments (Figure 2B). No polymorphism was detected for *K*_*AX_94741862* on 7AL in this population. Those markers closely linked to these QTL could be further detected in the ZM175/LX987 bi-parental population.

For the 3AL QTL, the most significant markers were located in the physical interval of 724,305,362–724,582,163 bp, where nine genes were annotated (Figures 2A,C). Sequence similarity analysis showed that *TraesCS3A01G499300*, annotated as a multidrug and toxic compound extrusion (MATE) family gene responsible for protein detoxification, had high amino acid sequence identity (87%) with OsPEZ2 (Tong et al., 2020). The gene *OsPEZ2* played an important role in metal uptake and translocation in rice and *pez2* mutant showed significantly reduced Fe concentration in root tips and xylem sap (Bashir et al., 2011; Ishimaru et al., 2011). *TraesCS3A01G499300* is 5,562 bp long and consists of seven exons in coding region (Figure 2C). Further sequence analysis revealed that *TraesCS3A01G499300* had abundant SNPs in its flanking and coding regions between ZM175 and LX987 (Supplementary Table 5), especially four missense SNPs within exons (Figure 2C), indicating *TraesCS3A01G499300* is likely to be the causal gene underlying 3AL QTL. Gene expression data indicate that the gene is constitutively expressed and is uniformly highly expressed in wheat root throughout developmental stages, which further supports its candidacy (Figure 2C).

## Discussion

### Effects of Marker Density and Linear Models on Wheat Genome-Wide Association Study

In previous GWAS studies on wheat Zn/Fe, large gaps in the genetic map were generally found due to the low marker number and density as well as relatively long LD decays. In this study, GWAS was conducted using high-density markers, such as 373,106 SNPs, making it possible to change the LD pattern, thereby resulting in efficient identification of MTAs in many low-recombination regions on wheat chromosomes (Kim and Yoo, 2016). Forty-one loci for GZnC/GFeC were detected, and the number is much higher than the QTL detected in previous literature (Gupta et al., 2021). Out of the 41 representative significant MTAs, only five markers came from the 90K SNP array, and 36 were from the 660K array, indicating that high-resolution GWAS was achieved by high density markers (Fu et al., 2020; Pang et al., 2020).

In terms of population size, a relatively small collection was used in this study. Nevertheless, the 166 core accessions were selected from over 400 elite cultivars and represented much diverse wheat accessions in China’s major wheat growing regions. The same population has been used to conduct GWAS on many yield or quality-related traits and performed highly informative in our previous studies (Liu et al., 2017; Zhai et al., 2018, 2020; Li et al., 2019; Fu et al., 2020). MLM using *Q* + *K* as covariant might be over-fitted possibly due to the strict control of population structure and kinship, leading to false negatives (Korte and Farlow, 2013). The multiple testing corrections tended to be too stringent to detect sufficient both major and minor MTAs. Whereas, the model FarmCPU as a complement, could avoid over-corrected population structure to some extent by utilizing fixed and random effect models iteratively (Kusmec and Schnable, 2018). Using three models together, reliable loci for GZnC and GFeC detected were almost doubled than using an MLM alone in the present research.

### Genetic Architecture Dissection by Comparing Current Loci With Known Quantitative Trait Locus/Loci

**FIGURE 3:**
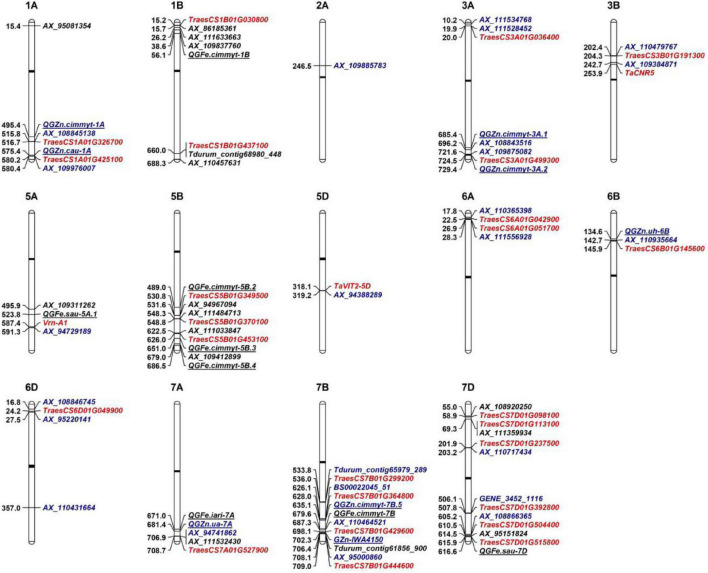
Genetic architectures of grain zinc and iron in wheat. The top of each graph indicates the chromosome number in wheat and the left shows the physical positions based on IWGSC RefSeq v.1.0. The loci identified to associate with GZnC and GFeC are shown with black and blue fonts, respectively. The representative markers (noted in Table 2) identified in this study were used to be compared with previous reported QTL that are underlined. The closest linked markers or mid-points of previous reported QTL intervals were from Tong et al. (2020). Promising candidate genes are highlighted as red color. Black bars indicate the locations of centromeres.

To date, hundreds of Fe/Zn-related QTL have been mapped on all wheat chromosomes except 6D by bi-parental linkage mapping and GWAS (Tong et al., 2020). For the 25 loci associated with GZnC in this study, eight have similar physical positions with known Zn-related QTL on 1AL (2), 3AL (2), 6BS, 7AL, and 7BL (2), respectively (Table 2 and Figure 3). As for GFeC, half of the 16 loci were identified to coincide with the documented QTL on 1BS, 5AL, 5BL (3), 7AL, 7BL, and 7DL, respectively. Major overlapping regions were observed on 5BL, where previous literatures reported a large number of Fe-related QTL by bi-parental mapping, confirming its importance for the gene discovery (Tong et al., 2020).

For the other 25 loci, comprising 17 loci for GZnC and 8 for GFeC, they were located in the chromosomal regions that were different from the QTL previously reported and are probably new loci (Table 2 and Figure 3). For example, three QTL on 6D (*AX_108846745*, 16.81 Mb; *AX_95220141*, 27.52 Mb; *AX_110431664*, 357.01 Mb) are highly likely to be novel since no Zn/Fe-related QTL has been previously mapped on this chromosome (Tong et al., 2020). Similarly, on chromosome 7D, there were only two Fe related QTL reported at 13.9 Mb and 616.6 Mb, so the three loci for GZnC (*AX_110717434*, 203.16 Mb; *GENE_3452_1116*, 506.11 Mb; and *AX_108866365*, 605.17 Mb) and two loci for GFeC (*AX_108920250*, 54.99 and *AX_111359934*, 69.31 Mb) were likely to be new. The new loci identified in this research have significantly enriched our understanding of the genetic basis of the complicated Zn and Fe traits in wheat.

### Promising Candidate Genes Related to Grain Zinc Concentration and Grain Iron Concentration

**FIGURE 4:**
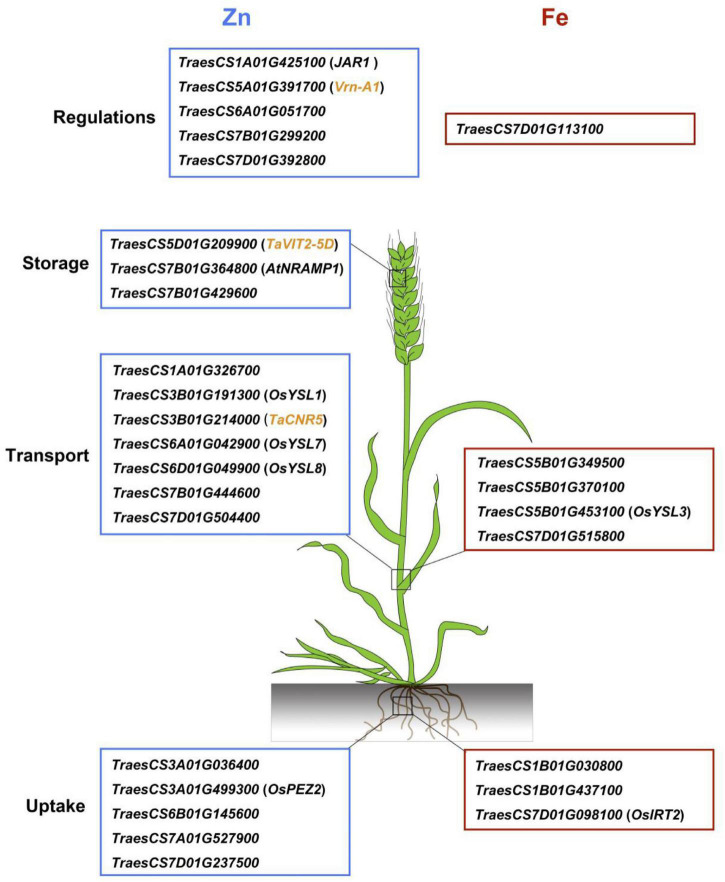
A brief model proposed for grain zinc and iron accumulation in common wheat based on the promising candidate genes identified. The wheat gene ID framed with blue and red boxes indicates zinc and iron genes, respectively, and the genes in the small brackets indicate known Zn/Fe related genes in model plants. The cloned zinc related genes in wheat are highlighted as yellow.

To date, numerous genes involved in Zn and Fe homeostasis in model plants have been extensively studied (Tong et al., 2020; Gupta et al., 2021). Wheat gene annotations from the IWGSC RefSeq v.1.0 provided a useful tool to further investigate the candidate genes located in or adjacent to the QTL identified. In the current study, 28 candidate genes were identified for Zn/Fe homeostasis, such as uptake, transport, storage, and regulations (Figure 4). The spatio-temporal expression profiles were highly consistent with their putative functions, indicating their possible roles in wheat Zn/Fe homeostasis. Of them, 11 genes were putative wheat orthologs of known *Arabidopsis* and rice genes related to Zn/Fe homeostasis, partially supporting the authenticity of the detected loci.

*TraesCS3A01G499300*, the candidate gene for 3AL QTL, was an ortholog of *OsPEZ2*, an efflux transporter for phenolics that served as chelators to facilitate metal uptake by root in rice (Bashir et al., 2011; Ishimaru et al., 2011). A very recent study demonstrated the pivotal roles of MATE family genes of protein detoxification for metal uptake and transport in rice roots and stems, further supporting the potential role of *TraesCS3A01G499300* on Zn/Fe homeostasis (Ren et al., 2021). Abundant sequence variations of the gene between ZM175 and LX987 were observed and provided strong evidence that it is possibly the causal gene and deserves further studies. In addition, we successfully developed KASP markers for the 7AL QTL but unfortunately it returned monomorphic in ZM175/LX987 RILs. However, it was interesting to find that the candidate gene *TraesCS7A01G527900* (706.91 Mb) underlying 7AL QTL was homologous with *TraesCS7B01G444600* (708.11 Mb) on 7BL and *TraesCS7D01G515800* (615.91 Mb) on 7DL for the three QTL identified in this study. The three candidate genes annotated as magnesium transporters were exactly the three homeologs for the same gene in each sub-genome. These loci are potentially important and we are now developing KASP markers for 7BL and 7DL QTL for validation. If any loci are validated in ZM175/LX987 or other mapping populations, the roles of all three loci in metal homeostasis can be presumed. The candidate genes identified in this study provided genetic bases for further elucidating the mechanisms of Zn and Fe homeostasis in wheat.

### Applications in Wheat Breeding for Biofortification

Since most common wheat had suboptimal GZnC and GFeC, it is imperative to identify Zn/Fe-related QTL or genes as many as possible and to re-introduce or to pyramid them into current wheat gene pools. In this study, 12 accessions with stable high GZnC or GFeC were identified with BLUE values over 50 mg/kg (Supplementary Figure 3). The elite germplasm, such as Xiaoyan54 and Xinong1376 are currently cultivars in wheat production and can be used immediately as donor parents for wheat biofortification.

Although no significant correlation was observed between TKW and GZnC/GFeC in the present study, one pleiotropic locus on 3AS (*AX_111528452*, 20.33 Mb) was identified to increase GZnC but decrease TKW in all four environments. This QTL may be useful in certain crosses for biofortification when TKW is high enough. In addition, when plant height does not significantly affect the overall performance, pleiotropic loci on 5AL (*AX_109311262*, 495.92 Mb) and 7BL (*Tdurum_contig61856_900*, 706.45 Mb) could exert effects on Zn/Fe enrichment. It should be noted that favorable allele frequencies for the above three representative markers were 0.31, 0.09, and 0.36, respectively, indicating quite low frequency in our association panel (Supplementary Table 3). Considering only very few GZnC/GFeC loci had negative pleiotropic effects on agronomic traits such as TKW and PH, we speculated that there were still high chances for the improvement of GZnC/GFeC without yield penalty (Velu et al., 2018).

The fast release of wheat reference genome sequences and pan-genomes will undoubtedly speed up the process of marker development and gene discovery for wheat biofortification. Cloning important genes involved in Zn/Fe uptake, transport, storage, and regulations and pyramiding favorable alleles are promising avenues to increase the GZnC and GFeC in wheat cultivars. The 3AL locus was validated using a bi-parental population and its candidate gene was proposed as an uptake related gene, highlighting its potential use in wheat breeding. High-throughput and breeder-friendly KASP markers will pave the way for MAS in breeding and accelerate the release of biofortified wheat.

## Conclusion

A GWAS using multiple models is demonstrated as a powerful approach for genetic dissection of micronutrient traits in wheat based on a high-resolution physical map. Sixteen loci were identified in the similar regions of known QTL related to Zn/Fe, and 25 loci were new. Twenty-eight promising candidate genes were identified based on bioinformatics analyses and gene expression data and are worthy of further investigation. The effect of one major QTL on 3AL was validated in a bi-parental population, highlighting its potential application for wheat biofortification.

## Data Availability Statement

The original contributions presented in the study are included in the article/Supplementary Material, further inquiries can be directed to the corresponding author/s.

## Author Contributions

YH and ZH conceived this work. YH and JT designed the experiments and wrote the manuscript. JT, CZ, MS, JS, DL, YZ, JZ, ZP, and LL completed the field work and data investigation. JT, CZ, and LF analyzed and interpreted the data. YH, ZH, AR, ML, and XX critically reviewed and revised the manuscript. All authors have read and approved the final manuscript.

## Conflict of Interest

The authors declare that the research was conducted in the absence of any commercial or financial relationships that could be construed as a potential conflict of interest.

## Publisher’s Note

All claims expressed in this article are solely those of the authors and do not necessarily represent those of their affiliated organizations, or those of the publisher, the editors and the reviewers. Any product that may be evaluated in this article, or claim that may be made by its manufacturer, is not guaranteed or endorsed by the publisher.

## Footnotes

1
http://www.capitalbiotech.com/

2
http://www.wheatgenome.org/

3
http://zzlab.net/GAPIT

4
https://github.com/YinLiLin/R-CMplot

5
http://www.uniprot.org/

6
http://wheat.cau.edu.cn/TGT/

7
http://wheat.cau.edu.cn/Wheat_SnpHub_Portal/

8
http://wheat-expression.com/

9
http://www.polymarker.info/
