# Supplementary material for: High Resolution Genome Wide Association Studies Reveal Rich Genetic Architectures of Grain Zinc and Iron in Common Wheat (Triticum aestivum L.)
Source: Front Plant Sci. 2022 Mar 16;13:840614. doi: 10.3389/fpls.2022.840614 (PMC8966881; doi:10.3389/fpls.2022.840614)
Supplement: Supplementary file 1 [file Data_Sheet_1.pdf]

Supplementary Figures

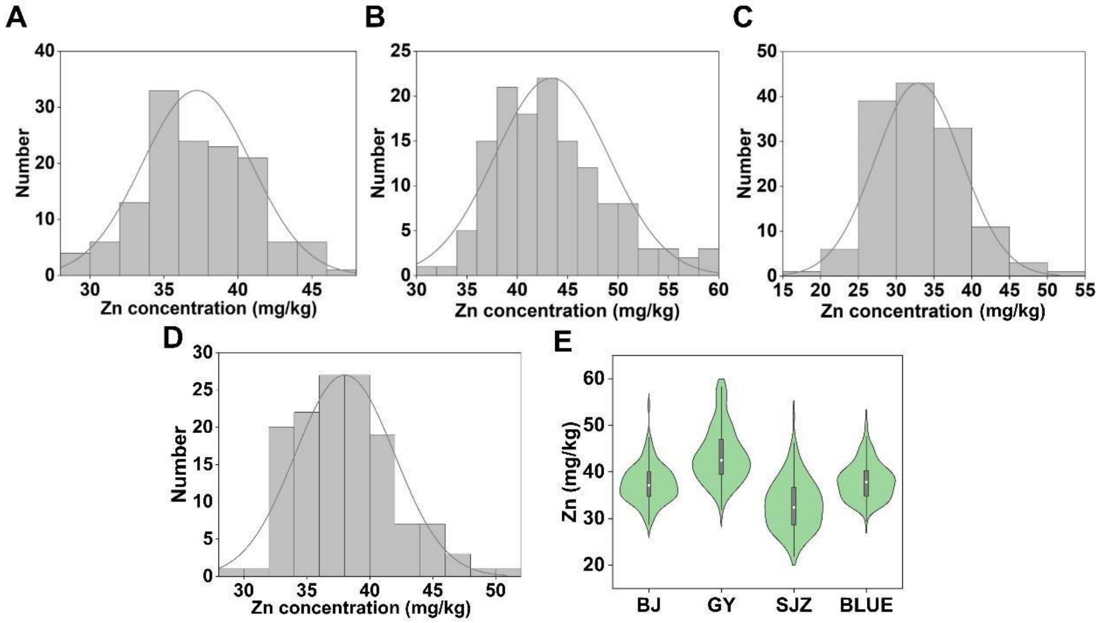

Figure S1 Phenotypic variations of GZnC in 166 accessions under different environments, including **(A)** Beijing, 2019–2020; **(B)** Gaoyi, 2019–2020; **(C)** Shijiazhuang, 2019–2020; and **(D)** BLUE. The violin plot for GZnC in these four environments was shown in **(E)**, allowing for comparison of the effect across locations.

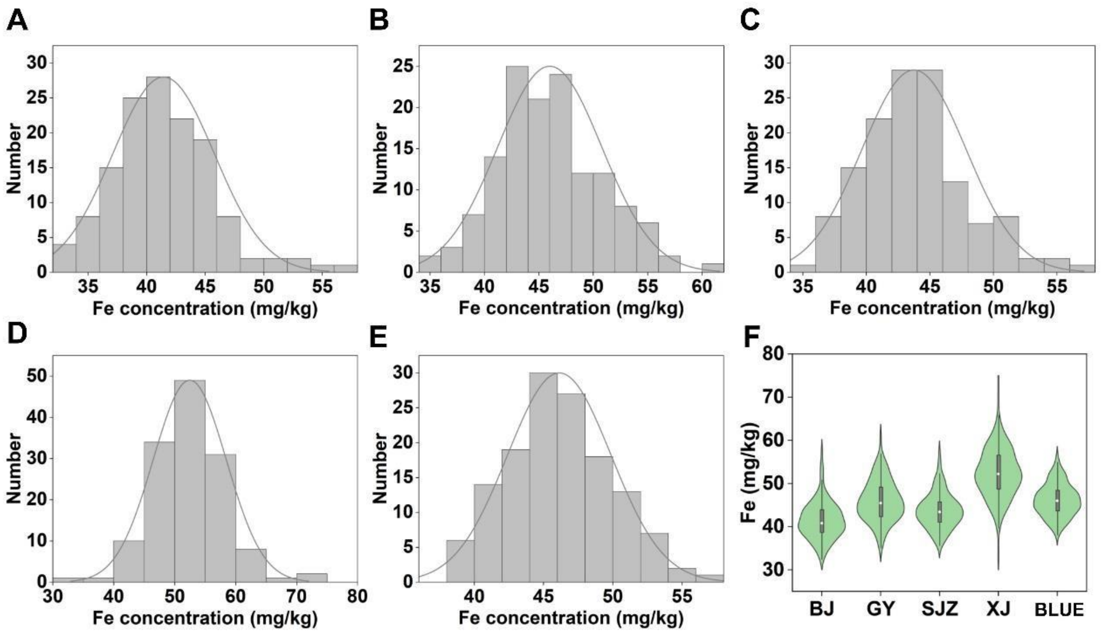

Figure S2 Phenotypic variations of GFeC in 166 accessions under different environments, including **(A)** Beijing, 2019–2020; **(B)** Gaoyi, 2019–2020; **(C)** Shijiazhuang, 2019–2020; **(D)** Xinjiang location, 2019–2020; and **(E)** BLUE. The violin plot for GFeC in these five environments was shown in **(F)**, allowing for comparison of the effect across locations.

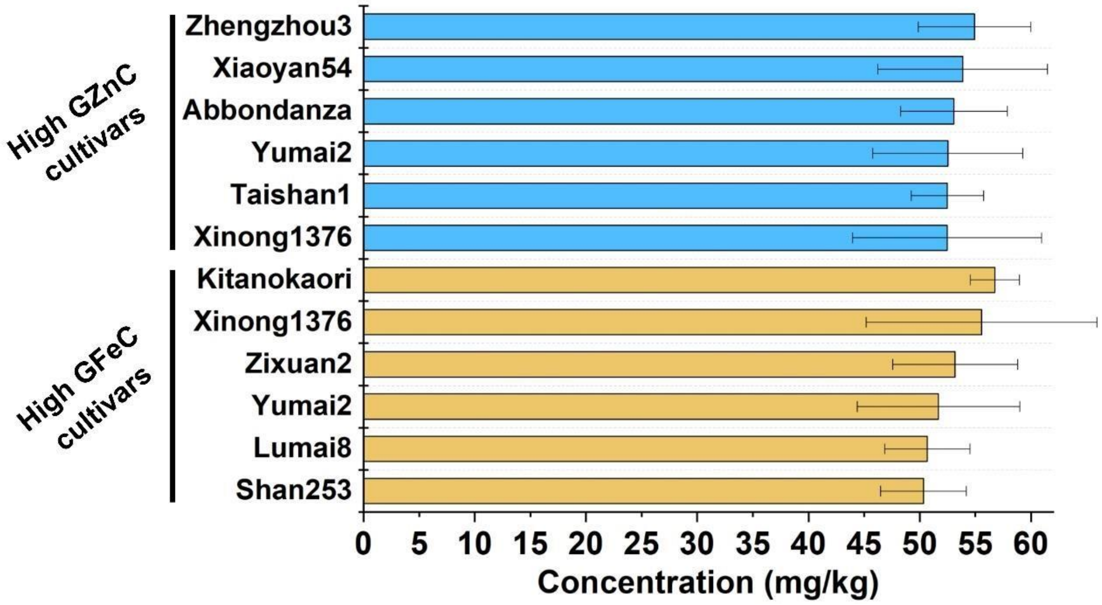

Figure S3 Twelve cultivars with stable high GZnC or GFeC in different environments. BLUE values were showed with standard derivations across different environments.

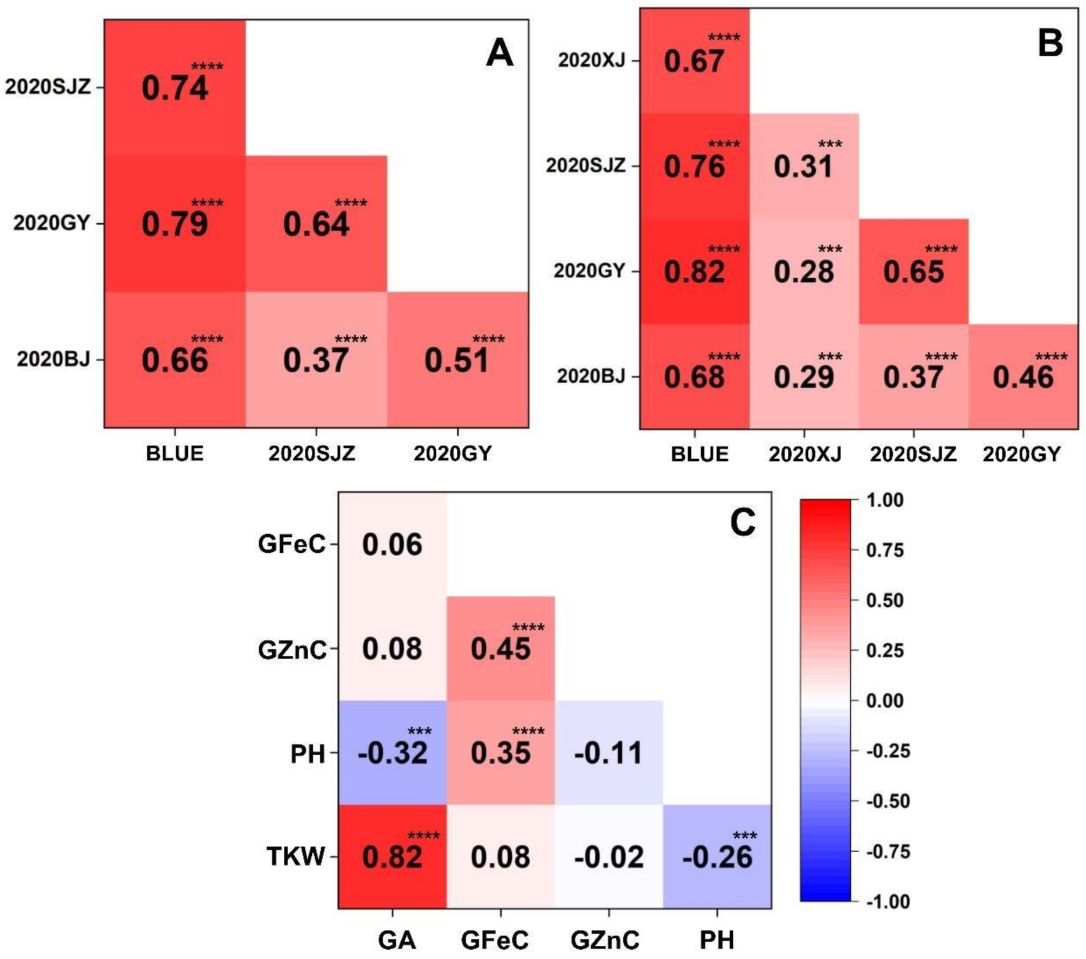

Figure S4 Pearson's correlation analyses among different environments for GZnC **(A),** GFeC **(B)**, and other agronomic traits based on BLUE values **(C)**. 20BJ, 20GY, 20SJZ, 20XJ: Beijing, Gaoyi, Shijiazhuang, and Xinjiang in 2019–2020. BLUE: best linear unbiased estimations. GZnC: grain zinc concentration; GFeC: grain iron concentration; TKW: thousand kernel weight; PH: plant height; GA: grain area. * $P < 0.05$; ** $P < 0.01$; *** $P < 0.001$; **** $P < 0.0001$

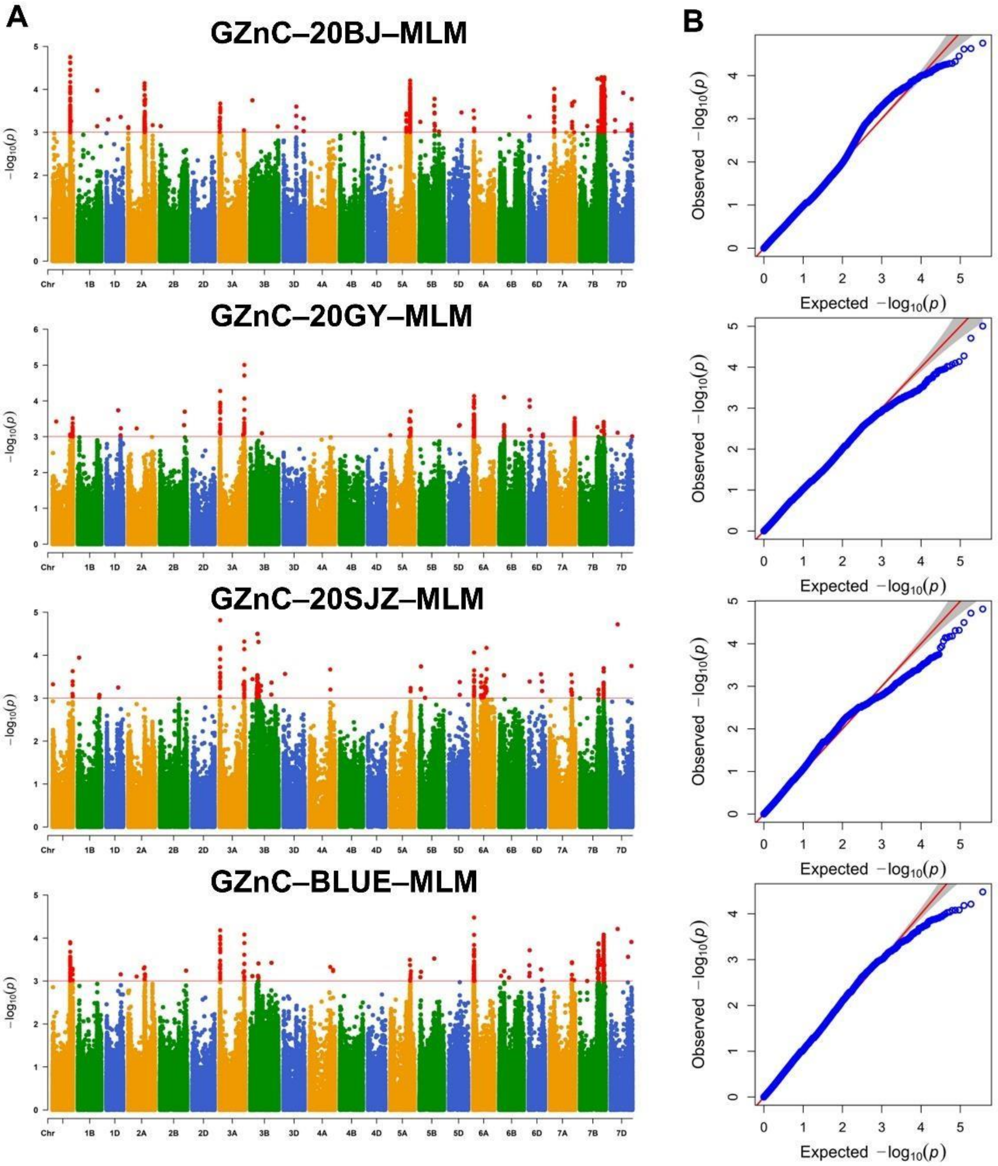

Figure S5 Part1

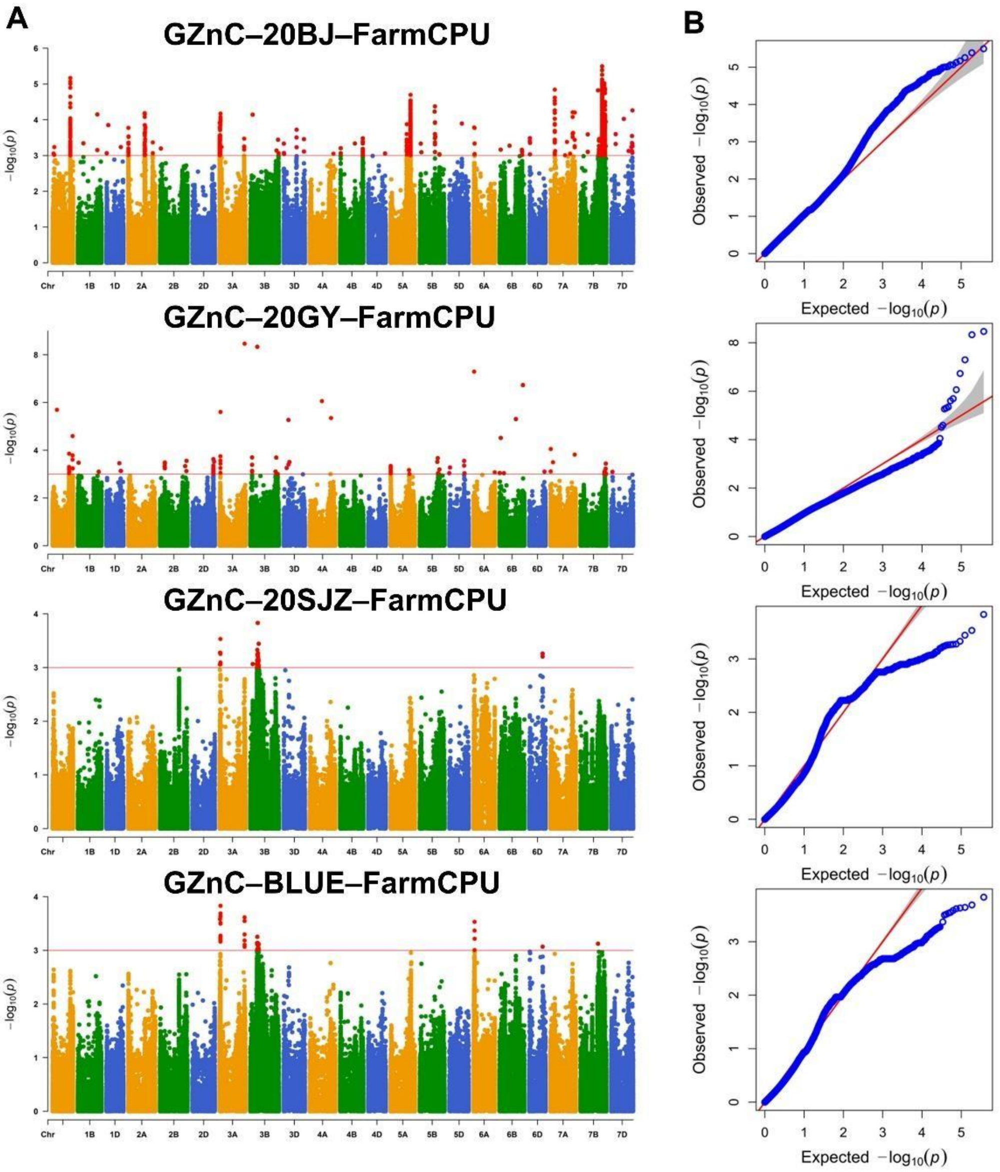

Figure S5 Part2

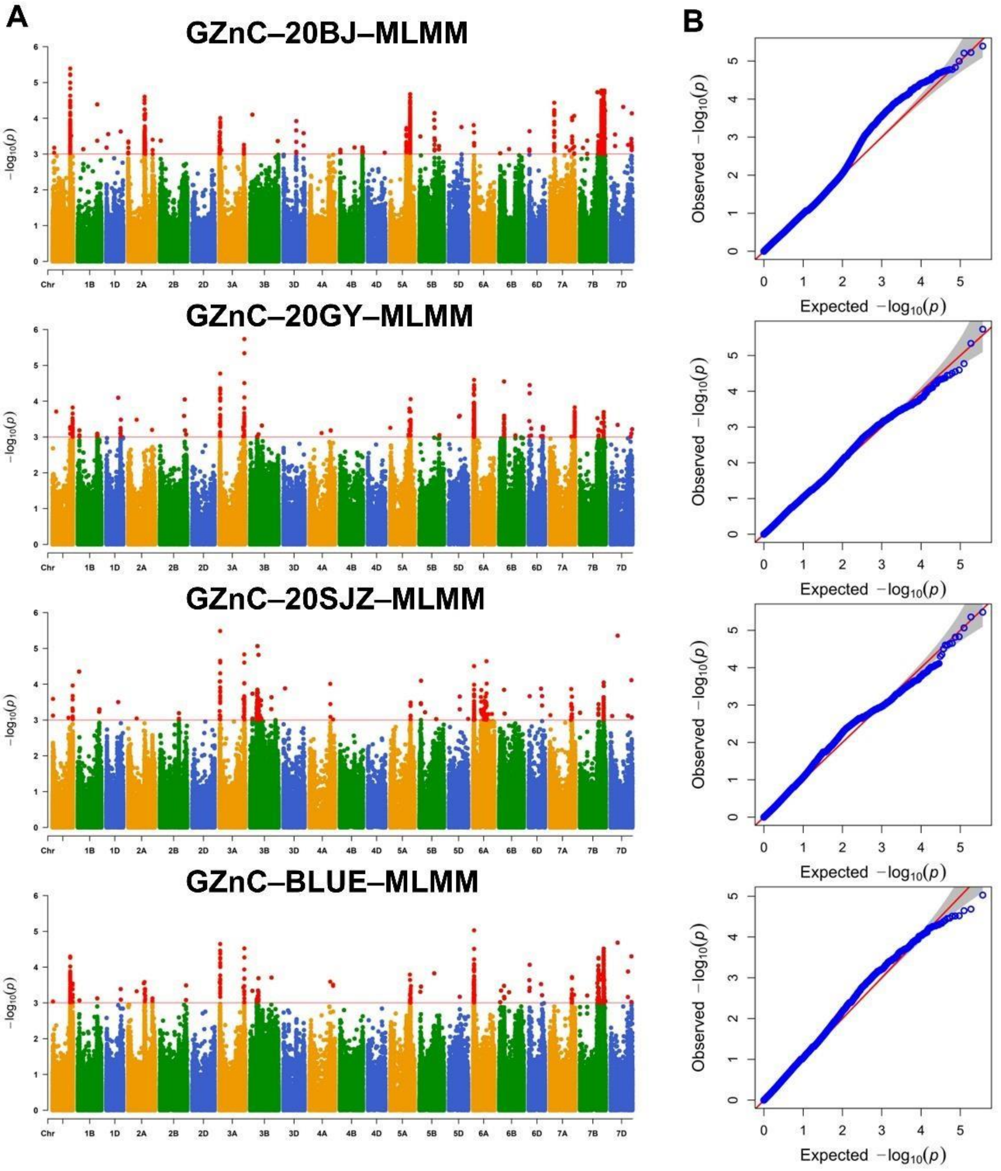

Figure S5 (A) Manhattan and **(B**) *Q*–*Q* plots for GZnC analyzed by MLM (part1), FarmCPU (part2), and MLMM (part3) in different environments. The threshold of $P = 1.0 \times 10^{-3}$ ($-\log_{10}(P) = 3.0$) was used for calling significant marker-trait associations (MTAs). GZnC: grain zinc concentration. 20BJ, 20GY, 20SJZ: Beijing, Gaoyi, and Shijiazhuang in 2019–2020. BLUE: the best linear unbiased estimations across environments.

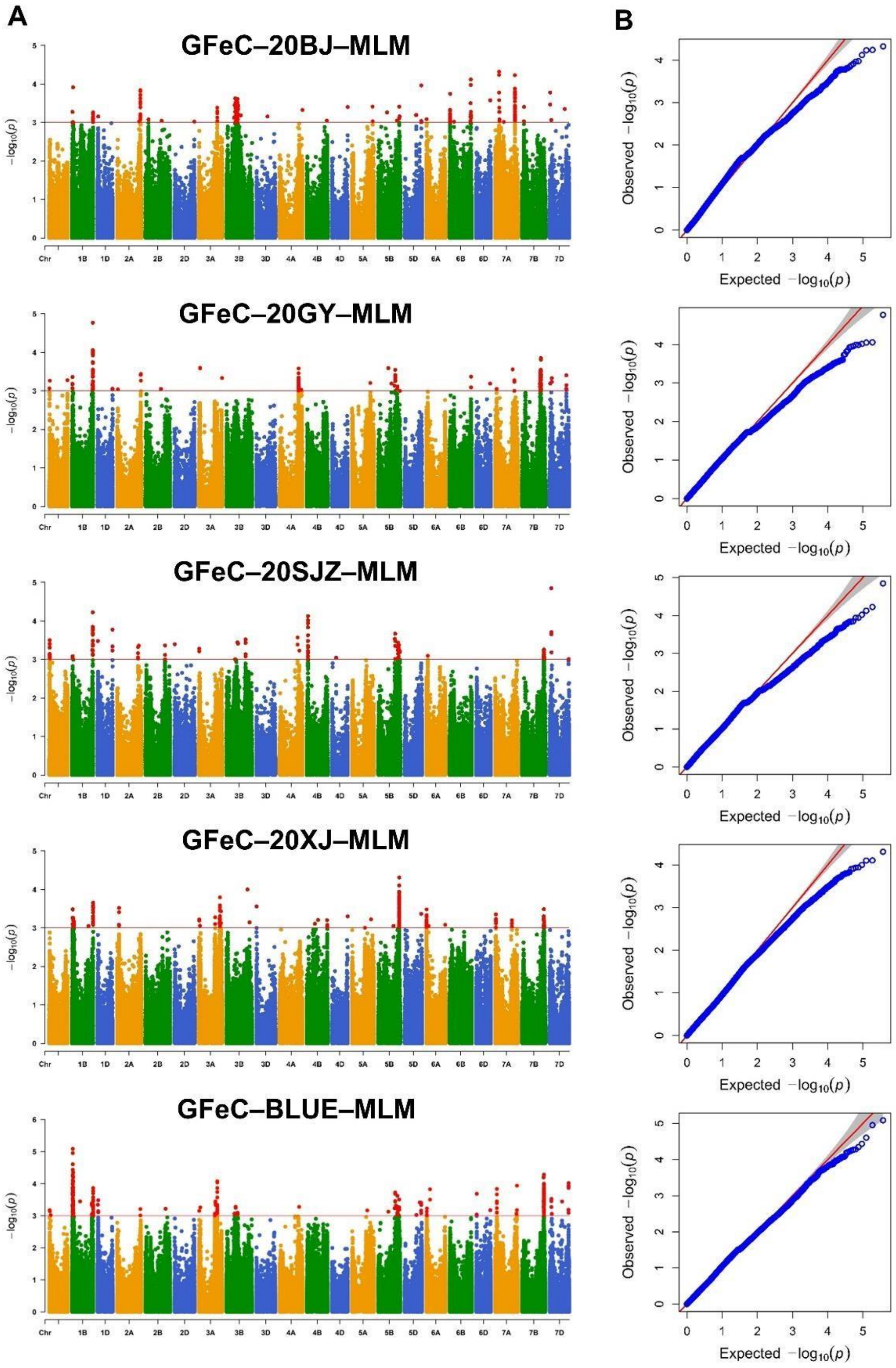

Figure S6 Part1

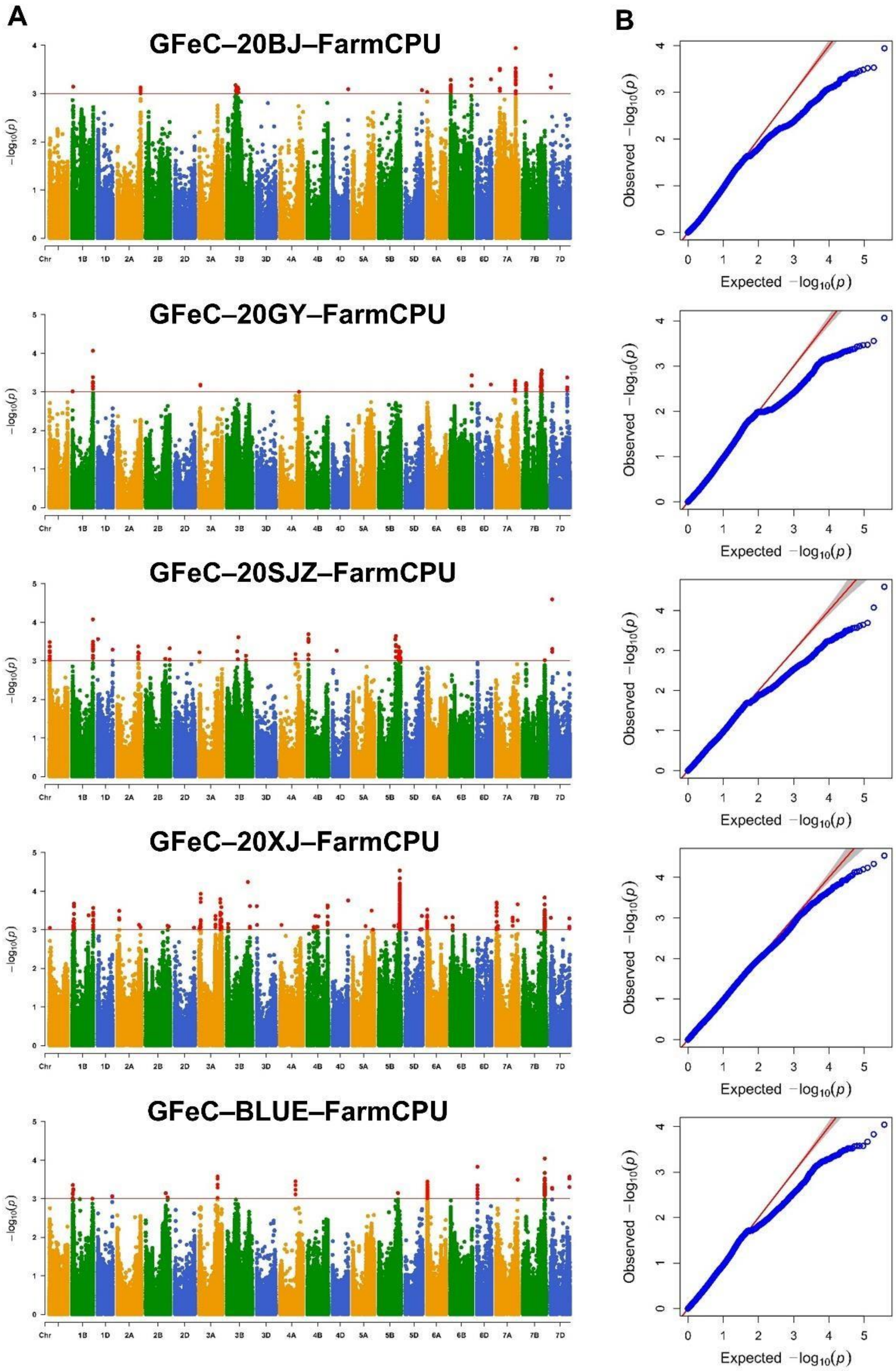

Figure S6 Part2

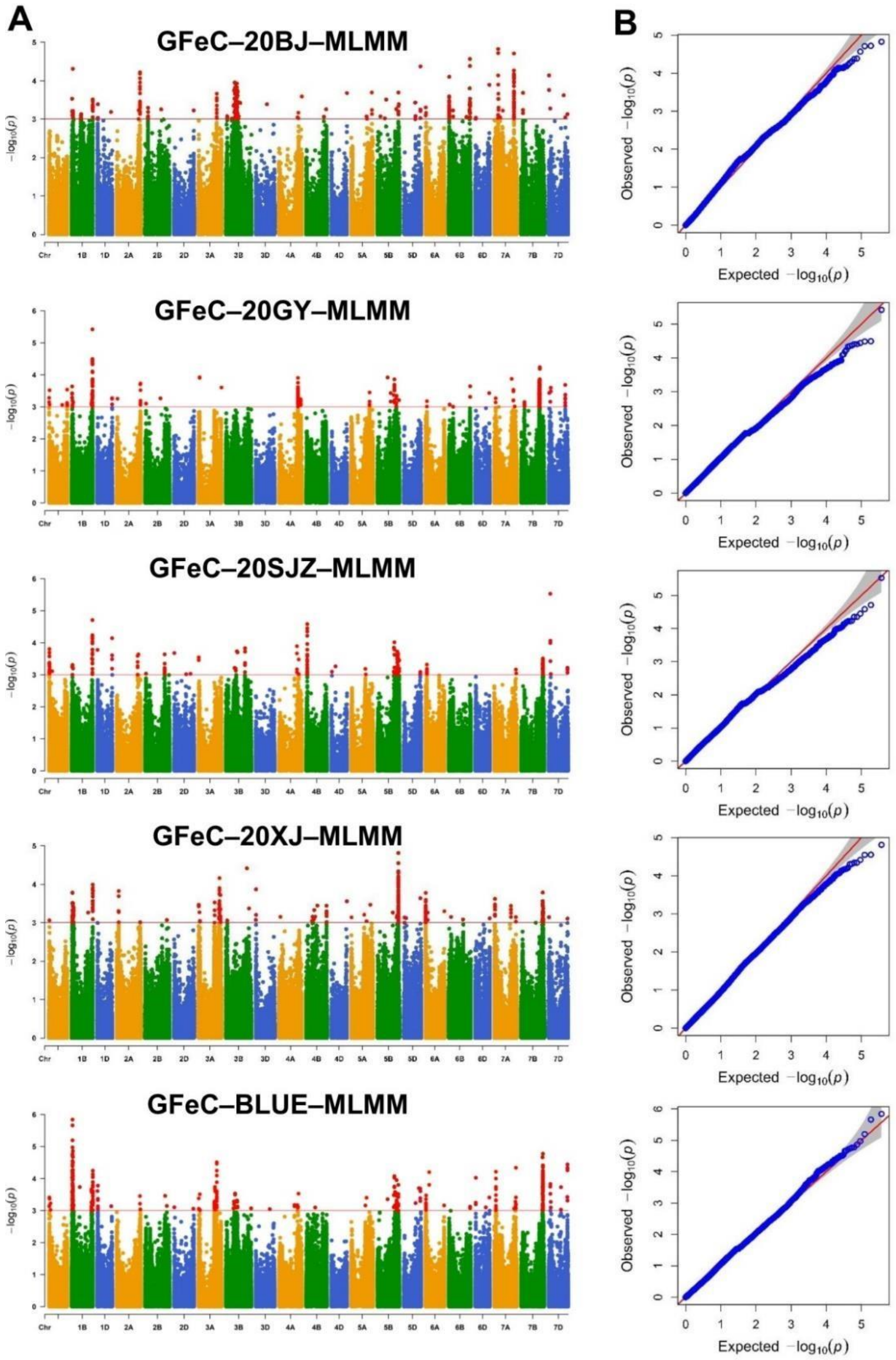

Figure S6 (A) Manhattan and **(B**) *Q*–*Q* plots for GFeC analyzed by MLM (part1), FarmCPU (part2), and MLMM (part3) in different environments. The threshold of $P = 1.0 \times 10^{-3}$ ($-\log_{10}(P) = 3.0$) was used for calling significant marker-trait associations (MTAs). GFeC: grain iron concentration. 20BJ, 20GY, 20SJZ, 20XJ: Beijing, Gaoyi, Shijiazhuang, and Xinjiang, 2019–2020. BLUE: the best linear unbiased estimations across environments.

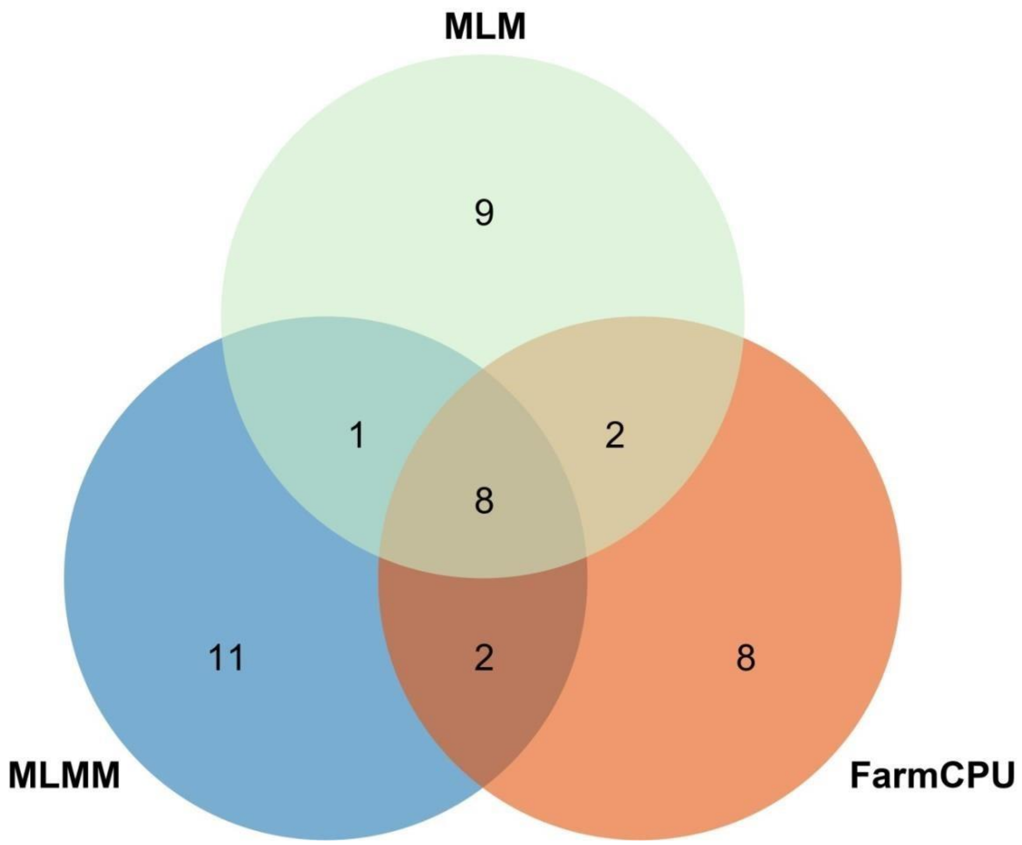

Figure S7 Venn diagram showing the number of stable loci associated with grain zinc 55 or iron concentrations detected with MLM, FarmCPU and MLMM.

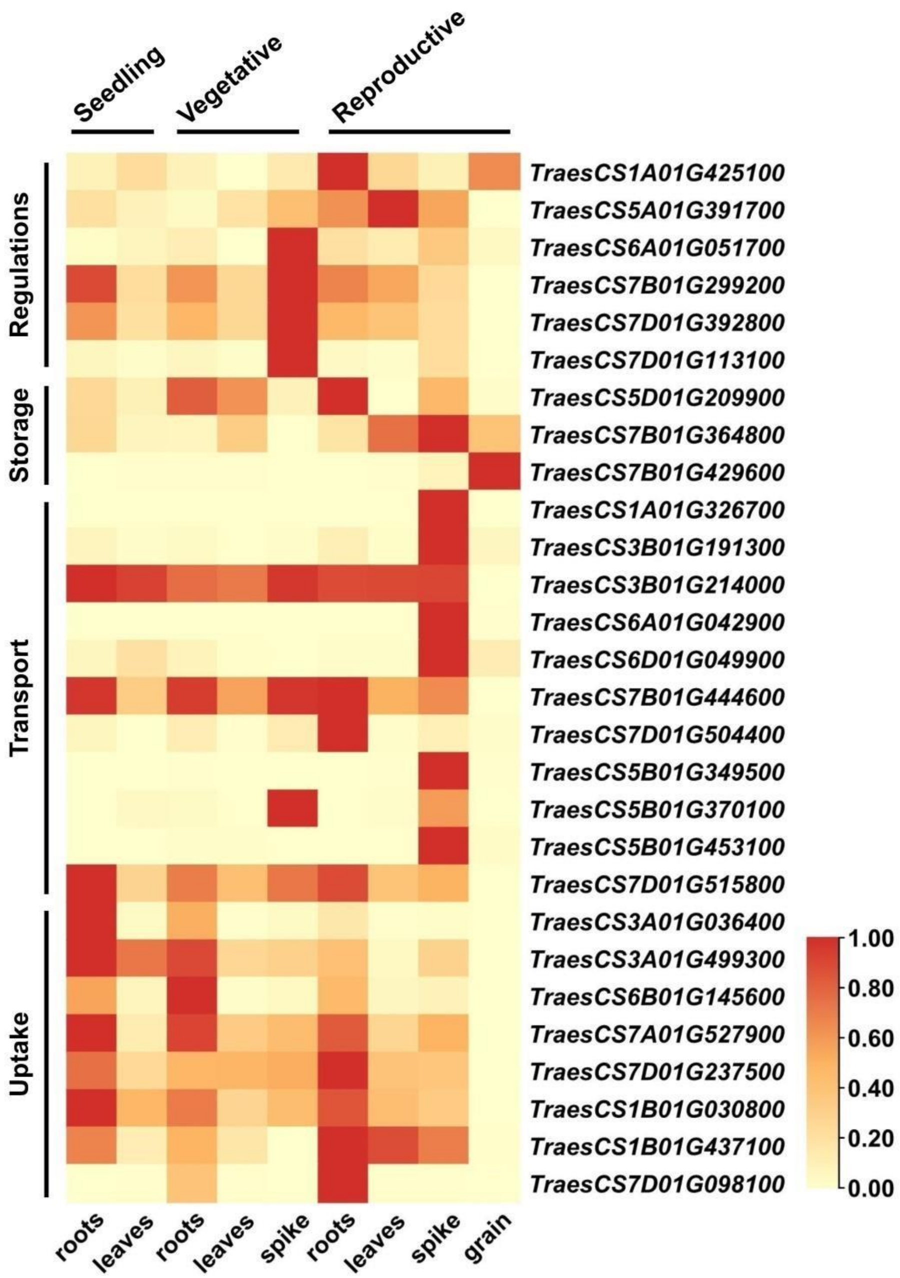

Figure S8 Heatmap showing the expression profiles of the candidate genes involved 60 in Zn/Fe uptake, transport, storage and regulations. Data were downloaded and 61 normalized from expVIP database (http://wheat-expression.com/).
